# In depth comparisons of neuropsychological & neuroimaging profile of primary psychiatric disorders and behavioral variant FTD

**DOI:** 10.1002/alz.089445

**Published:** 2025-01-03

**Authors:** Agnès Denève, Thibaud Lebouvier, Renaud Lopes, Gregory Kuchcinski, Florence Pasquier, Ali Amad, Maxime Bertoux

**Affiliations:** ^1^ University of Lille ‐ Inserm, Lille, Hauts‐de‐France France; ^2^ Univ. Lille, Inserm, CHU Lille, Lille Neuroscience & Cognition, UMR‐S 1172, Lille, Hauts‐de‐France France; ^3^ INSERM U1171 / Neuroradiology department, University Hospital, Lille France; ^4^ University of Lille, Inserm, CHU Lille U1172, Lille, Hauts‐de‐France France; ^5^ Univ Lille CHU Inserm LilleNcog, DISTAlz, LiCEND, CNRMAJ, Lille France; ^6^ Lille Neuroscience & Cognition, Inserm, Univ. Lille, CHU Lille, LiCEND & DistALZ, Lille France

## Abstract

**Background:**

Over the past years, social cognition has been envisaged as a promising domain to distinguish behavioral variant frontotemporal degeneration (bvFTD) from its main differential diagnoses that is primary psychiatric disorders (PPD). The core‐processes approach, which has emphasized the importance of emotion recognition and mentalizing, has been particularly useful to better characterize each condition and enhance the earliness of FTD’s diagnosis. However, new findings evidencing conflicting results regarding the ability of social cognition to distinguish bvFTD from PPD have underlined the importance of moving beyond the core processes approach.

**Method:**

We reviewed all cases with a suspission of bvFTD in the last 8 years in the Lille memory clinic, at least followed‐up for 24 months with a neuropsychological assessment and an MRI and/or PET‐scan. We then applied a quantitative comparison approach based on total scores, then a qualitative approach, based on responses and errors types. Then, neuroimaging analyses were run, and biomarkers were analyzed.

**Result:**

Data of 56 patients with a probable to certain bvFTD and 47 patients with a primary psychiatric disorders (late major depression, bipolar disorders, schizophrenia…) were analyzed at classical and social cognitive (mini‐SEA) tests. Overall, clinical groups were not different on executive functionning, attention, motor & perceptual functions. Minor differences were retrieved in memory and langage processing. Important differences were retrieved in social cognition. Qualitative differences were retrieved in facial emotion recognition (inter & intra valence errors) and mentalizing (type of mental inference, emotional labelling), as well as memory functioning (primacy/recency ratio, intrusions). Anatomical and functional brain networks involved showed a combination of overlaping and distincts areas. Regarding biomarkers, NFLs showed promising results with AUC = 0.88).

**Conclusion:**

While the usual approach (considering general or subscores scores) may not be the more efficient way, a more qualitative neuropsychological approach has the potential to provide relevant cognitive markers for the clinical distinction between bvFTD and PPD, particularly regarding social cognition.

